# Immunoadsorption and Plasma Exchange are Comparable in Anti-Neutrophil Cytoplasmic Antibodies or Anti-Glomerular Basement Membrane Removal Kinetics

**DOI:** 10.1016/j.ekir.2024.06.031

**Published:** 2024-06-27

**Authors:** Marion Sallee, Noémie Resseguier, Thomas Crepin, Daniel Bertin, Dominique Bertrand, Mickaël Bobot, Thierry Krummel, Nicolas Maillard, Julie Moussi-Frances, Marion Pelletier, Pascale Poullin, Cédric Rafat, Thomas Robert, Benjamin Terrier, Lionel Rostaing, Stanislas Faguer, Noémie Jourde-Chiche

**Affiliations:** 1 AP-HM, Hôpital de la Conception, Centre de Néphrologie et Transplantation Rénale, Marseille, France; 2 Aix-Marseille Univ, C2VN, INSERM, INRAE, Marseille, France; 3 Aix-Marseille Univ, CEReSS/UR 3279 - Health Services and Quality of Life Research, Marseille, France; 4 APHM, Methodological Support Unit for Clinical and Epidemiological Research, Marseille, France; 5 CHU de Besançon, Service de Néphrologie, Besançon, France; 6 AP-HM, Hôpital de la Timone, Laboratoire d’Immunologie, Marseille, France; 7 CHU de Rouen, Service de Néphrologie, Rouen, France; 8 CHU de Strasbourg, Service de Néphrologie, Strasbourg, France; 9 CHU de Saint-Etienne, Service de Néphrologie, Saint-Etienne, France; 10 Hôpital Saint-Joseph, Service de Néphrologie, Marseille, France; 11 AP-HM, Hôpital de la Conception, Service d’Aphérèses, Marseille, France; 12 AP-HP, Hôpital Tenon, Service de Néphrologie, Paris, France; 13 AP-HP, Hôpital Cochin, Service de Médecine interne, Paris, France; 14 CHU de Grenoble, Service de Néphrologie, Grenoble, France; 15 CHU de Toulouse, Service de Néphrologie, Toulouse, France

**Keywords:** ANCA-associated vasculitis, anti-GBM disease, antibody removal, immunoadsorption, plasma exchanges

## Abstract

**Introduction:**

Apheresis allows the fast removal of autoantibodies in anti-glomerular basement membrane (anti-GBM) disease, and in severe antineutrophil cytoplasmic antibodies (ANCA)-associated vasculitis. The CINEVAS study tested whether immunoadsorption (IA) allowed a faster removal of ANCA and/or anti-GBM antibodies than plasma exchanges (PEx).

**Methods:**

CINEVAS was a prospective multicenter study comparing IA to PEx in consecutive patients with ANCA and/or anti-GBM vasculitides. The primary objective was the reduction rate in autoantibody titers between the beginning of the first and the end of the seventh apheresis session. Secondary objectives were number of sessions needed to obtain desired reduction rates; reduction rates of total Ig levels; tolerance of sessions; and patients’ outcome.

**Results:**

The results of 38 patients (16 treated with IA and 22 with PEx), and 43 autoantibodies, were analyzed. There was no difference in the reduction rates in autoantibody titers between IA and PEx over 7 sessions (respectively 98% vs. 96%, *P* = 0.39). The numbers of sessions needed to obtain undetectable autoantibodies, or 50%, 75%, or 90% reductions, did not differ between techniques. Greater reduction rates of autoantibodies were observed when plasma was separated by filtration compared to centrifugation, with IA and PEx. IA allowed a greater reduction in total IgG levels, and better preservation of total IgA and IgM levels than PEx. PEx sessions required higher volumes of plasma, IA sessions higher volumes of citrate; IA sessions were longer.

**Conclusions:**

IA and PEx were comparable in ANCA or anti-GBM removal kinetics, despite a faster reduction in total IgG with IA.

## Graphical abstract

**Figure:**
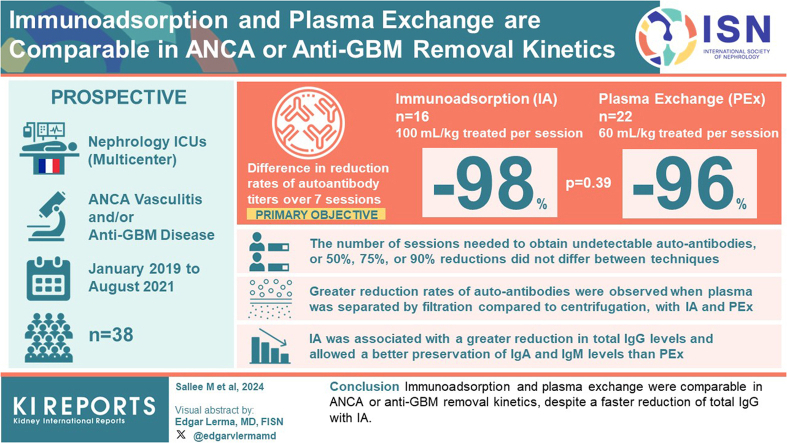

ANCA and anti-GBM antibodies may have a direct pathogenic role in ANCA-associated vasculitides and anti-GBM disease. Rapid elimination of ANCA and/or anti-GBM antibodies by different techniques of apheresis may reduce patients’ exposure to these autoantibodies and improve functional and vital outcomes. In patients with ANCA-associated vasculitides, despite the negative results of the PEXIVAS trial,^1^ PEx can be proposed in patients with a high risk of kidney failure according to the recent international recommendations.^2^^,^^3^ PEx are also proposed to patients with both ANCA and anti-GBM antibodies, who were excluded from the PEXIVAS trial.^1^^,^^4^^,^^5^ PEx are also highly recommended in patients with anti-GBM disease presenting with intraalveolar hemorrhage and/or if renal damage does not already requires dialysis.^4^^,^^5^ However, PEx are associated with several drawbacks: the limited volume of plasma treated per session, the absence of specificity for Ig epuration, with a loss of coagulation factors possibly favoring bleeding complications, the necessity to infuse a substitution solute. IA, which is broadly used in renal transplantation, could be more specific for IgG elimination, with larger volumes of plasma treated per session, without substitution solute.^6^ Only 1 multicenter study^7^ has compared the kinetics of elimination of ANCA between IA and PEx in 44 patients with renal involvement, which showed no difference on renal outcome after 6 months. The objective of the CINEVAS study was to test whether IA allowed a faster removal of ANCA and/or anti-GBM antibodies in patients with vasculitides.

## Methods

CINEVAS (NCT03635385) was a prospective multicenter nonrandomized trial comparing IA to PEx in terms of autoantibody removal kinetics.

Patients were recruited in nephrology intensive care units in 8 centers in France between January 2019 and August 2021. This study was conducted in accordance with the principles of the declaration of Helsinki and the International Conference on Harmonisation Guidelines for Good Clinical Practice. All patients gave their written informed consent before any study-related procedure.

Patients included were adults (aged >18 years), had a diagnosis of vasculitis (new or relapsing) associated with ANCA (either anti-proteinase 3 [PR3] or anti-myeloperoxidase [MPO]) or with anti-GBM antibodies, were about to be treated with corticosteroids and either cyclophosphamide or rituximab, and had an indication for apheresis according to the investigator. Patients were excluded if they were pregnant or breastfeeding, had a vasculitis without positive ANCA or anti-GBM antibodies, had severe anemia (hemoglobin level < 70 g/l), or changed apheresis technique before the third session.

The primary end point was the reduction rate in autoantibody titers between the beginning of the first apheresis session and the end of the seventh session. Secondary end points were: the mean reduction rate in autoantibody levels par session; the number of sessions needed to obtain undetectable autoantibodies, or to reduce their levels by 50%, 75% or 90%; the removal kinetics according to the specificity of the autoantibody (anti-PR3, -MPO or -GBM); the kinetics of total IgG, IgM, and IgA removal; the kinetics of platelets and fibrinogen; the tolerance of sessions; and the clinical outcome (patient survival, renal survival, estimated glomerular filtration rate, Birmingham Vasculitis Activity Score [BVAS]) at day 15, day 30, 6 months, and 12 months.

Patients were treated with a minimum of 7 apheresis sessions, either IA or PEx (the same technique for a given patient), as per the center preference. The coordinating center (AP-HM, Marseille), which routinely used both techniques, aimed to balance groups. Plasma was separated from blood by centrifugation or by filtration (no double filtration cascade). IA was performed with Globaffin columns (Fresenius Medical Care), and the volume of plasma treated per session was 100 ml/kg of body weight, without substitution solute, as per the manufacturer’s instruction. The volume of plasma treated by PEx session was 60 ml/kg of body weight, with substitution with 4% or 5% albumin, possibly associated with fresh frozen plasma if needed for coagulation purposes.

Blood samples were drawn at the beginning and at the end of each session, to measure the following parameters: anti-PR3, anti-MPO, anti-GBM antibodies; total IgG, IgM and IgA levels; platelets and hemoglobin levels; and fibrinogen level. These parameters were also measured at day 15, day 30, 6 months, and 12 months, together with serum creatinine and C-reactive protein levels. There was no centralization of blood samples, and values were compared for a given patient in the same local laboratory. Autoantibody levels were considered negative if they were below the positivity threshold of the local laboratory. Glomerular filtration rate was estimated with the modification of diet in renal disease formula. Kidney failure was defined as the need for renal replacement therapy, of estimated glomerular filtration rate <15 ml/min per 1.73 m^2^. Vasculitis activity was evaluated with the BVAS at the inclusion and at day 30, 6 months, and 12 months. Vasculitis damage was evaluated by the vasculitis damage index at day 30, 6 months, and 12 months.

Baseline clinical, biological, and therapeutic characteristics of the patients were first described and compared according to IA or PEx groups. Qualitative characteristics were described as numbers and percentages and compared using chi-square test if valid (Fisher exact test, otherwise). Quantitative characteristics were described as medians (1st quartiles–3rd quartiles) and compared using Mann-Whitney U test given the sample sizes. End points were analyzed by considering patients and/or autoantibodies as statistical units. When autoantibodies were considered as statistical units, clustered estimates and clustered statistical tests were used to take into account the correlation that may exist between autoantibodies within patients with 2 autoantibodies. The reduction rate in autoantibody levels was described and compared according to IA or PEx groups. The description was based on medians (1st quartiles–3rd quartiles), and the comparison was based on Mann-Whitney U test given the sample sizes. The same analysis was performed according to centrifugation or filtration groups.

Patients’ outcomes were described according to IA or PEx groups, using numbers and percentages. BVAS was described and compared according to IA or PEx groups. The description was based on medians (1st quartiles–3rd quartiles), and the comparison was based on Mann-Whitney U test given the sample sizes. Technical parameters related to apheresis sessions were described and compared according to IA or PEx groups. The description was based on medians (1st quartiles–3rd quartiles), and the comparison was based on Mann-Whitney U test, given the distribution of the data.

The number of apheresis sessions needed to obtain 50%, 75%, 90% reductions, and undetectable autoantibodies (in patients for whom the reduction occurred) was described and compared according to IA or PEx groups. The description was based on medians (1^st^ quartiles–3^rd^ quartiles), and the comparison was based on Mann-Whitney U test given the sample sizes.

The reduction rate in total IgG, IgM, and IgA levels was described and compared according to IA or PEx groups. The description was based on medians (1^st^ quartiles–3^rd^ quartiles), and the comparison was based on Mann-Whitney U test, given the sample sizes. The same analysis was performed according to centrifugation or filtration groups.

All statistical analyses were performed using R version 4.2.2 (R Foundation for Statistical Computing, Vienna, Austria). The survey R package was used to analyze data when autoantibodies were considered as the statistical units. All tests were 2-sided. Statistical significance was set at a threshold of *P* < 0.05.

## Results

### Patients

Forty patients were included in the CINEVAS study in 8 university hospitals in France: 18 patients were treated with IA, and 22 patients were treated with PEx. Among them, 1 patient was excluded because he had negative autoantibodies despite a clinical presentation typical of ANCA-associated vasculitis, and 1 patient was excluded because of a change of technique after the 2 first sessions. Thus, results from 38 patients were analyzed (16 treated with IA, and 22 with PEx). Baseline characteristics of these 38 patients are summarized in Table 1. Some patients had 2 different types of auto-antibodies, and overall, the participants had 43 autoantibodies; 15 patients were anti-MPO positive, 15 were anti-PR3 positive, 3 were anti-GBM positive, 3 were anti-MPO and anti-GBM double positive, 1 was anti-PR3 and anti-GBM double positive, and 1 had both anti-MPO and anti-PR3 antibodies.

**Table 1:** Baseline characteristics of the 38 patients

Characteristics	IA *N*= 16	PEx *N*= 22	*P-*value
Male	4 (25.0)	15 (68.2)	0.0086
Female	12 (75.0)	7 (31.8)	
Age, yr	57 (52–70)	64 (57–74)	0.22
Weight, kg	68.5 (62.8–75.9)	74 (70–80.6)	0.15
Height, cm	167 (161–170)	175 (167–178)	0.023
BMI, kg/m^2^	25 (23–27)	25 (24–27)	0.81
Diabetes	1 (6.25)	3 (14.3)	0.62
Hypertension	6 (40.0)	8 (38.1)	0.91
Current smoking	2 (14.3)	10 (58.8)	0.029
Dyslipidemia	2 (13.3)	1 (5.3)	0.57
Coronary artery disease	0 (0)	2 (9.5)	0.50
Stroke	3 (18.75)	1 (4.8)	0.30
History of cancer	2 (12.5)	4 (20.0)	0.67
1st flare of vasculitis	14 (87.5)	22 (100)	0.17
Vasculitis relapse	2 (12.5)	0 (0)	
Undergoing dialysis at diagnosis	8 (50.0)	5 (22.7)	0.08
Dialysis or serum creatinine ≥ 500 μmol/l	13 (81.25)	10 (45.5)	0.026
Serum creatinine, μmol/l	549 (400–662)	467 (335–591)	0.37
eGFR, ml/min per 1.73 m^2^	7 (6–11)	10 (7–16)	0.17
Urinary output, ml/d	1700 (1225–1950)	1600 (1500–1850)	1.00
Intraalveolar hemorrhage	3 (18.7)	7 (31.8)	0.37
Anti-PR3 +	7 (43.7)	10 (45.5)	0.92
Anti-MPO +	7 (43.7)	12 (54.6)	0.51
Anti-GBM +	5 (31.2)	2 (9.1)	0.11
Corticosteroids	16 (100)	22 (100%)	1.0
i.v. methylprednisolone	11 (68.8)	15 (68.2)	0.97
rituximab	8 (50.0)	16 (72.7)	
i.v. cyclophosphamide	8 (50.0)	8 (36.4)	0.43
Plasma filtration	7 (43.7)	9 (40.9)	0.86
Plasma centrifugation	9 (56.3)	13 (59.1)	

BMI, body mass index; eGFR, estimated glomerular filtration rate; GBM, glomerular basement membrane; IA, immunoadsorption; MPO, myeloperoxidase; PEx, plasma exchange; PR3, proteinase 3.

Data are expressed as *n* (%) or median (Q1–Q3).

### Autoantibody Removal Kinetics

**Figure 1:**
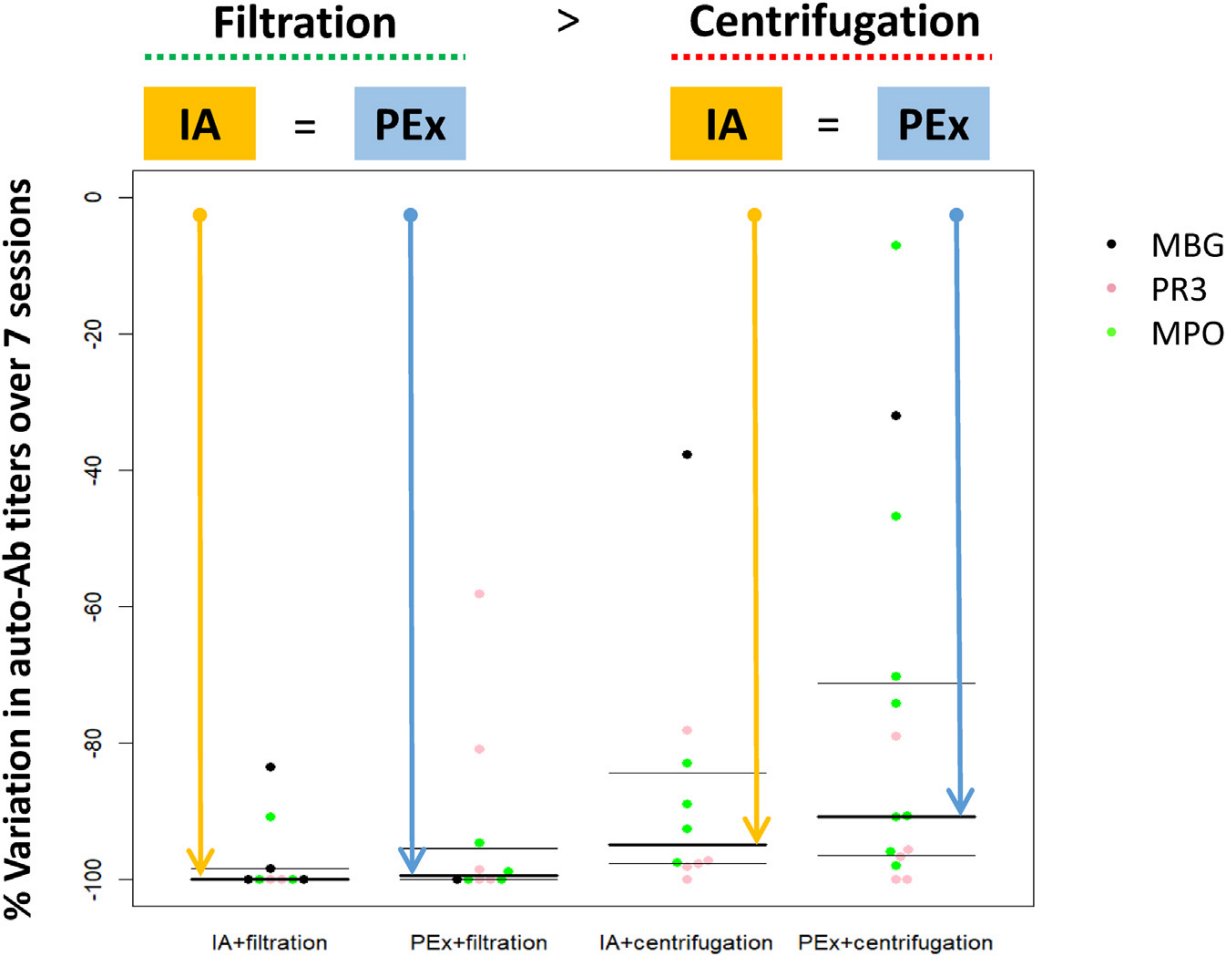
Percent reduction of autoantibody titers (*n* = 43) over 7 sessions of apheresis, with immunoadsorption (IA) or plasma exchange (PEx), after plasma separation by filtration or by centrifugation. Ab, antibody; GBM, glomerular basement membrane; MPO, myeloperoxidase; PR3, proteinase 3.

Removal kinetics of autoantibodies are detailed in Table 2. There was no significant difference in the reduction rates of autoantibodies between IA and PEx between the beginning of the 1st session and the end of the 7th session, whether this was compared by considering patients (Figure 1) or antibodies (Table 2). There was no difference between IA and PEx in the reduction rates over 7 sessions for any of the anti-PR3, anti-MPO, and anti-GBM antibodies (Table 2). There was also no difference between IA and PEx when considering the autoantibody reduction levels, compared to baseline, at the start of each apheresis session (Supplementary Table S1).

**Table 2:** Comparison of the reduction rates in autoantibody levels between immunoadsorption (IA) and plasma exchanges (PEx), considering all autoantibodies, and specifically anti-MPO, anti-PR3 or anti-GBM antibodies

Auto-antibodies and sessions	IA	PEx	*P*-value
All autoantibodies	*n* = 19	*n* = 24	
Over 7 sessions	98 (90–100) (*n* = 19)	96 (78–100) (*n =* 24)	0.39
Session #1 (*n =* 31)	42 (5–70) (*n =* 16)	53 (41–62) (*n =* 15)	0.56
Session #2 (*n =* 37)	70 (55–83) (*n =* 17)	52 (21–65) (*n =* 20)	0.035
Session #3 (*n =* 34)	75 (67–84) (*n =* 16)	53 (24–62) (*n =* 18)	0.028
Session #4 (*n =* 35)	68 (56–84) (*n =* 16)	67 (38–71) (*n =* 19)	0.42
Session #5 (*n =* 34)	76 (62–88) (*n =* 14)	57 (32–66) (*n =* 20)	0.054
Session #6 (*n =* 32)	77 (58–94) (*n =* 15)	52 (32–66) (*n =* 17)	0.031
Session #7 (*n =* 32)	67 (56–81) (*n =* 13)	65 (38–73) (*n =* 19)	0.36
Anti-MPO antibodies	*n =* 7	*n =* 12	
Over 7 sessions	93 (90–99)	93 (73–98)	0.58
Anti-PR3 antibodies	*n =* 7	*n =* 10	
Over 7 sessions	98 (97–100)	98 (85–100)	0.65
Anti-GBM antibodies	*n =* 5	*n =* 2	
Over 7 sessions	98 (84–100)	66 (32–100)	0.84

GBM, glomerular basement membrane; IA, immunoadsorption; MPO, myeloperoxidase; PEx, plasma exchange; PR3, proteinase 3.

The number of sessions needed to obtain 50%, 75% or 90% reductions or undetectable autoantibodies, among patients who reached these objectives within the 7 apheresis sessions, did not differ between IA and PEx (Supplementary Table S2).

Of note, whatever the apheresis technique (IA or PEx), plasma separation by filtration was associated with a higher reduction rate in antibody levels than separation by centrifugation (Figure 1 and Table 3). The results in autoantibody reduction levels after 15 days, 30 days, 6 months, and 12 months are provided in Supplementary Table S3.

**Table 3:** Comparison of the reduction rate in autoantibody levels between centrifugation and filtration techniques of plasma separation, considering all autoantibodies (*n =* 43) and specifically anti-MPO, anti-PR3 and anti-GBM antibodies

Auto-antibodies and sessions	Centrifugation	Filtration	*P*-value
All antibodies	*n =* 24	*n =* 19	
Over 7 sessions (*n =* 43)	92 (77–98) (*n =* 24)	100 (96–100) (*n =* 19)	0.0019
Session 1 (*n =* 31)	43 (7–74) (*n =* 17)	55 (44–66) (*n =* 14)	0.66
Session 2 (*n =* 37)	52 (16–68) (*n =* 20)	66 (52–75) (*n =* 17)	0.11
Session 3 (*n =* 34)	49 (22–75) (*n =* 21)	71 (60–81) (*n =* 13)	0.032
Session 4 (*n =* 35)	65 (38–73) (*n =* 19)	69 (59–73) (*n =* 16)	0.38
Session 5 (*n =* 34)	61 (42–69) (*n =* 22)	77 (53–100) (*n =* 12)	0.097
Session 6 (*n =* 32)	49 (28–63) (*n =* 19)	71 (66–100) (*n =* 13)	0.00066
Session 7 (*n =* 32)	60 (30–71) (*n =* 20)	75 (59–100) (*n =* 12)	0.036
Anti-MPO antibodies	n = 12	*n =* 7	
Over 7 sessions	90 (73–93)	100 (97–100)	0.0045
Anti-PR3 antibodies	*n =* 10	*n =* 7	
Over 7 sessions	97 (96–100)	100 (90–100)	0.45
Anti-GBM antibodies	*n =* 2	*n =* 5	
Over 7 sessions	35 (32–38)	100 (98–100)	0.07

GBM, glomerular basement membrane; IA, immunoadsorption; MPO, myeloperoxidase; PEx, plasma exchange; PR3, proteinase 3.

Results are presented as median (Q1–Q3).

### Removal of Total Igs

Removal kinetics of total IgG, IgM, and IgA levels are presented in Supplementary Table S4. The reduction rate of total IgG was higher with IA than with PEx, particularly during the first 4 sessions. Overall, IgG levels were similar between IA and PEx after 7 sessions (considering that some patients treated with PEx received fresh frozen plasma). Total IgM and IgA levels were significantly more preserved with IA than with PEx, from the first session to the seventh, and the levels of total IgM and total IgA remained higher after the seventh session in patients treated with IA compared to PEx.

There was no difference in the reduction rates of total IgG, IgM, and IgA according to the plasma separation technique (centrifugation or filtration) (Supplementary Table S5).

### Tolerance and Clinical Outcome

There was no significant difference between techniques in the reduction rates of platelet counts (37% [53–12] with IA vs. 36% [43–22] with PEx, *P* = 0.99), or fibrinogen levels (61% [71–58] with IA vs. 76% [80–63] with PEx, *P* = 0.07) over 7 apheresis sessions.

Adverse events and technical issues with both techniques are presented in Table 4. As expected, the volume of plasma treated per IA session was almost twice that of PEx. The volume of citrate was also larger with IA than PEx. IA sessions were longer than PEx sessions.

**Table 4:** Adverse events and technical parameters with immunoadsorption (IA) and plasma exchanges (PEx)

Outcome and technical parameters	IA	PEx	*P*-value
Patients’ outcome	*N=*16 patients	*N=*22 patients	
Death < M12	3 (of sepsis, septic shock, or respiratory failure)	0	0.066
Serious infections (nonfatal)	1 zoster	1 SARS-COV2 infection	>0.99
Serious bleeding	5 (4)	6 (6)	>0.99
(requiring transfusion)			>0.99
Poor tolerance of apheresis session	7 episodes in 5 patients	2 episodes in 2 patients	0.11
Apheresis sessions	*n =* 112 sessions Median (Q1–Q3)	*n =* 154 sessions Median (Q1–Q3)	*P*
Duration, minutes	224 (189–272)	104 (93–122)	6.2e-10
Plasma volume treated, l	6.5 (5.9–7)	4 (4.3–3.4)	1.1e-10
P. volume treated, ml/kg	98 (89–99)	53 (47–56)	1.75e-10
Citrate infusion volume, ml	662 (432–1282)	296 (237–418)	1.5e-05
Plasma infusion volume, ml	0 (0–0)	3415 (573–3882)	1.7e-06
Fibrinogen infusion dose, g	0 (0–0)	0 (0–0)	0.94

IA, immunoadsorption; PEx, plasma exchange.

The evolution of BVAS, and the proportion of patients in remission over time (BVAS = 0), did not differ between techniques (Supplementary Table S6). Three patients treated with IA died (1 with anti-GBM antibody, 2 with anti-MPO antibodies, all 3 on dialysis at diagnosis) after 2.5, 3, and 4 months respectively. No death was observed in the PEx group. In the IA group, among the 8 patients requiring dialysis at inclusion, 3 died, and 5 remained on dialysis; 1 additional patient required temporary dialysis at day 15 and subsequently recovered kidney function. In the PEx group, among the 5 patients requiring dialysis at inclusion, 3 recovered kidney function; and 2 additional patients reached chronic kidney failure (1 on dialysis). Among the 8 patients on dialysis at 12 months, initial autoantibodies were as follows: 2 anti-GBM positive, 2 double positive anti-GBM and anti-MPO, 1 double positive anti-GBM and anti-PR3, 2 anti-MPO, and 1 anti-PR3.

## Discussion

This prospective nonrandomized study shows no difference in the reduction rates of ANCA or anti-GBM antibodies over 7 sessions of IA versus PEx in patients with ANCA vasculitis or anti-GBM disease receiving an induction therapy with corticosteroids and cyclophosphamide and/or rituximab. Autoantibody reduction rates exceeded 90% with both techniques. Despite the greater reduction in total IgG levels with IA, the specific reduction in anti-MPO, anti-PR3, or anti-GBM antibody titers was not significantly greater. As expected, volumes of plasma treated were larger with IA than with PEx, sessions were longer with IA than with PEx, and the requirement for plasma infusion was lower with IA than with PEx. Higher volumes of citrate infusion were used with IA than with PEx. Interestingly, whatever the apheresis technique, the reduction rates of autoantibody levels were greater if plasma had been separated by filtration compared to centrifugation.

To our knowledge, only 2 studies compared the efficacy of PEx and IA in rapidly progressive crescentic glomerulonephritis^7^ and in ANCA vasculitis.^8^ In the prospective randomized study of Stegmayr *et al.*,^7^ 44 patients were included to compare clinical efficacy of IA versus PEx. Renal outcome was described as nondifferent at 6 months between IA and PEx. Unfortunately, very few data on apheresis techniques were available, and no data were provided on ANCA or anti-GBM antibody reduction kinetics. The second is a retrospective study comparing IA and PEx in anti-MPO ANCA vasculitis in 48 patients.^8^ The authors reported an advantage for IA in terms of anti-MPO titers reduction after 1 month (81% with IA vs. 53% with PEx); however, not all patients had the same number of sessions, there was a lot of missing data in this retrospective study (reduction rates available in only 17/28 patients from the PEx group), and the impact of immunosuppressive therapies could also have played a role on autoantibody levels at this more distant time point. In addition, not all patients had the same number of sessions. In the CINEVAS study, the primary end point was the percent reduction of autoantibody titers over 7 apheresis sessions (which were performed over a maximum period of 14 days), allowing for greater comparability.

The superiority of plasma separation by filtration in CINEVAS is another interesting result. A randomized, prospective, paired, crossover study conducted in 21 patients^9^ compared filtration (membrane-based) to centrifugation (centrifuge-based) for the separation of plasma in therapeutic plasma exchange performed for various indications. Total IgG removal, and total IgM removal, were similar between techniques. A longer duration of sessions, and a greater loss of platelets, were observed with plasma membrane-filtration, favoring the use of centrifugation. Similarly, no difference was found in CINEVAS between filtration and centrifugation in the removal of total IgG, IgM, or IgA. Nevertheless, plasma separation by filtration resulted in a greater removal of autoantibodies. This discrepancy between total IgG removal and autoantibody removal, which mirrors the absence of benefit of IA for autoantibody removal despite a greater removal of total IgG, warrants further research.

Patients’ outcome was a secondary objective of the CINEVAS study; however, the interpretation of results warrants caution because the groups were unbalanced at inclusion, with more severe patients in the IA group. Patients treated with IA were more likely to require dialysis or display a serum creatinine above 500 μmol/l at inclusion. In addition, among the 7 patients with positive anti-GBM antibodies, 5 were in the IA group. Patients with anti-GBM disease or those who have a double positivity for ANCA and anti-GBM antibodies have a poorer renal prognosis than patients with ANCA antibodies only.^10^ Dialysis at diagnosis is a major factor of poor kidney survival in anti-GBM disease.^11^^,^^12^

To conclude, the CINEVAS study shows no benefit of IA over PEx in the removal kinetics of anti-MPO, anti-PR3, or anti-GBM antibodies, despite higher volumes of plasma treated and greater reduction in total IgG levels. Plasma separation by filtration was associated with a greater reduction in autoantibody levels compared to centrifugation, despite similar removal rates of total IgG. When apheresis is considered in a patient with ANCA vasculitis and/or anti-GBM disease, both apheresis techniques (IA and PEx) can be used, and plasma separation by filtration may be favored.

## Disclosure

NJC, BT, and SF received speaking and expertise fees from CSL VIFOR. All the other authors declared no competing interests.

## Supplementary Material

Supplementary File (PDF)

**Table S1.** Comparison of reduction rates in autoantibody levels at the beginning of each apheresis session, compared to the beginning of the first apheresis, between immunoadsorption (IA) and plasma exchanges (PEx).

**Table S2.** Number of apheresis sessions needed to obtain 50%, 75%, or 90% reductions or undetectable autoantibodies, among patients who reached these objectives within 7 sessions.

**Table S3.** Comparison of the reduction rates in autoantibody levels between immunoadsorption (IA) and plasma exchanges (PEx), considering all autoantibodies, and specifically anti-MPO, anti-PR3 or anti-GBM antibodies, during the follow-up at 15 days, 30 days, 6 months, and 12 months.

**Table S4.** Comparison of reduction rates in total IgG, IgM, and IgA levels between immunoadsorption (IA) and plasma exchanges (PEx), over 7 sessions and per session.

**Table S5.** Comparison of reduction rates in total IgG, IgM, and IgA levels between plasma separation techniques (centrifugation or filtration) over 7 sessions.

**Table S6.** Evolution of the Birmingham Vasculitis Activity Index (BVAS) over time, in patients treated with immunoadsorption (IA) or plasma exchanges (PEx).
