# Supplementary material for: Immunoadsorption and Plasma Exchange are Comparable in Anti-Neutrophil Cytoplasmic Antibodies or Anti-Glomerular Basement Membrane Removal Kinetics
Source: Kidney Int Rep. 2024 Jun 27;9(9):2767–73. doi: 10.1016/j.ekir.2024.06.031 (PMC11403088; doi:10.1016/j.ekir.2024.06.031)
Supplement: Supplementary File (PDF) [file mmc1.pdf]

Supplemental Material

Supplementary Table 1: Comparison of reduction rates in auto-antibody levels at the beginning of each apheresis session, compared to the beginning of the first apheresis, between immunoadsorption (IA) and plasma exchanges (PEx).

	IA	PEx	p
All auto-antibodies	n= 19	n=24	
Session 1 (n=43)	0 (n=19)	0 (n=24)	-
Session 2 (n=39)	47 [0-55] (n=19)	43 [15-51] (n=20)	0.96
Session 3 (n=38)	58 [18-75] (n=17)	56 [23-68] (n=21)	0.54
Session 4 (n=38)	65 [42-79] (n=17)	66 [20-79] (n=21)	0.96
Session 5 (n=35)	73 [59-82] (n=14)	74 [25-82] (n=21)	0.58
Session 6 (n=34)	75 [66-86] (n=15)	78 [27-90] (n=19)	0.93
Session 7 (n=32)	74 [53-87] (n=13)	73 [42-90] (n=19)	0.98

Supplementary Table 2. Number of apheresis sessions needed to obtain 50%, 75% or 90% reductions or undetectable auto-antibodies, among patients who reached these objectives within 7 sessions.

Objective	Median	Q1-Q3	p
50% reduction			0.73
IA (n=18)	2	1-3.75	
PEx (n=22)	2	2-3.75	
75% reduction			0.87
IA (n=16)	4	2-6	
PEx (n=18)	3	3-43	
90% reduction			0.78
IA (n=10)	5.5	4.25-6.75	
PEx (n=14)	5	5-6	
Undetectable			0.59
IA (n=6)	5	4-7	
PEx (n=9)	6	5-7	

Supplementary Table 3: Comparison of the reduction rates in auto-antibody levels between immunoadsorption (IA) and plasma exchanges (PEx), considering all auto-antibodies, and specifically anti-MPO, anti-PR3 or anti-GBM antibodies, during the follow-up at 15 days, 30 days, 6 months and 12 months.

	IA	PEx	p
All auto-antibodies	n= 19	n=24	
D15 (n=28)	82 [79-93] (n=14)	77 [52-95] (n=14)	0.52
D30 (n=29)	81 [64-99] (n=16)	59 [37-91] (n=13)	0.17
M6	100 [55-100] (n=9)	100 [57-100] (n=13)	0.47
M12	97 [64-100] (n=8)	93 [64-100] (n=14)	0.47
Anti-MPO antibodies	n= 7	n=12	
D15 (n=13)	80 [70-82] (n=6)	70 [47-95] (n=7)	0.83
D30 (n=13)	66 [60-69] (n=7)	36 [9-52] (n=6)	0.18
M6 (n=8)	56 [33-100] (n=3)	20 [11-98] (n=5)	0.45
M12 (n=11)	78 [39-100] (n=4)	70 [32-88] (n=7)	0.50
Anti-PR3 antibodies	n=7	n=10	
D15 (n=10)	85 [80-100] (n=5)	80 [73-88] (n=5)	0.40
D30 (n=12)	95 [86-100] (n=6)	92 [81-95] (n=6)	0.29
M6 (n=10)	100 [100-100] (n=3)	100 [98-100] (n=7)	0.26
M12 (n=8)	97 [94-100] (n=2)	100 [97-100] (n=6)	0.82
Anti-GBM antibodies	n=5	n=2	
D15 (n=5)	94 [87-97] (n=3)	56 [36-76] (n=2)	0.8
D30 (n=4)	99 [37-100] (n=3)	19 (n=1)	1
M6 (n=4)	85 [54-100] (n=3)	100 (n=1)	1
M12 (n=3)	93 [87-100] (n=2)	97 (n=1)	1

Supplementary Table 4. Comparison of reduction rates in total IgG, IgM and IgA levels between immunoadsorption (IA) and plasma exchanges (PEx), over 7 sessions and per session. *Results are presented as median [Q1-Q3].*

	IA	PEx	p
Total IgG removal			
Over 7 sessions	95 [91-96]	88 [77-94]	**0.036**
Session #1	73 [55-77]	33 [21-58]	**0.0006**
Session #2	74 [59-80]	50 [55-64]	**0.0039**
Session #3	71 [57-74]	55 [18-66]	**0.015**
Session #4	67 [63-72]	59 [30-65]	**0.016**
Session #5	66 [63-71]	62 [52-66]	0.24
Session #6	64 [49-72]	56 [26-62]	0.30
Session #7	64 [53-73]	53 [27-67]	0.23
Total IgM removal			
Over 7 sessions	59 [44-67]	89 [74-93]	**0.0030**
Session #1	14 [0-17]	53 [11-64]	0.057
Session #2	15 [3-22]	20 [0-57]	0.45
Session #3	21 [13-29]	40 [17-60]	0.096
Session #4	25 [12-26]	50 [23-62]	**0.017**
Session #5	22 [0-25]	60 [50-70]	**0.0001**
Session #6	15 [0-20]	50 [32-67]	**0.0004**
Session #7	17 [0-25]	33 [0-50]	0.32
Total IgA removal			
Over 7 sessions	52 [46-61]	88 [72-92]	**0.0013**
Session #1	7 [0-21]	27 [18-62]	**0.0044**
Session #2	12 [5-21]	52 [22-63]	**0.0018**
Session #3	14 [11-22]	55 [21-65]	**0.0094**
Session #4	17 [13-25]	60 [24-61]	**0.019**
Session #5	10 [8-25]	60 [50-67]	**0.0002**
Session #6	10 [4-19]	50 [35-67]	**0.0003**
Session #7	17 [10-20]	50 [33-67]	**0.0033**

Supplementary Table 5: Comparison of reduction rates in total IgG, IgM and IgA levels between plasma separation techniques (centrifugation or filtration) over 7 sessions. *Results are presented as median [Q1-Q3].*

	**Centrifugation** **n = 24**	**Filtration** **n = 19**	p
Total IgG removal			
Over 7 sessions	93 [90-95]	89 [74-95]	0.22
Total IgM removal			
Over 7 sessions	80 [61-90]	60 [44-71]	0.072
Total IgA removal			
Over 7 sessions	63 [53-91]	61 [45-78]	0.43

Supplementary Table 6. Evolution of the Birmingham Vasculitis Activity Index (BVAS) over time, in patients treated with immunoadsorption (IA) or plasma exchanges (PEx).

	IA N=16 patients	PEx N=22 patients	p
BVAS	Median [Q1-Q3]	Median [Q1-Q3]	
Inclusion	18.5 [12-22.3]	16 [14-20]	0.84
Day 15	8 [6-12]	12 [11.5-12.5]	0.12
Day 30	8 [8-12]	11 [8-12.8]	0.64
6 months	4 [0-9]	0 [0-7]	0.51
12 months	0 [0-8]	0 [0-2.5]	0.52
